# 

**Published:** 1907-12

**Authors:** 


					﻿Sixty=third Year.
Vol. LX1II. DECEMBER, 1907.	5
BUFFALO
MEDICAL JOURNAL
Established 1845 by AUSTIN FLINT M. D.
L ii A.V V	EDITOR:
WARREN POTTER, M. D.
DFC-? ^€7	!
■	> ASSISTANT EDITORS:
‘ WILLIAM C. KRAUSS, M.D.	NELSON W. WILSON, M. D.
--------ASSOCIATE EDITORS;
JAMES WRIGHT PUTNAM, M. D. ERNEST WENDE, M. D.
JOHN PARMENTER, M. D.	JOHN A. MILLER, Ph. D.
MAUD J. FRYE, M. D.
				

